# Hashimoto’s thyroiditis increases the risk of new-onset systemic lupus erythematosus: a nationwide population-based cohort study

**DOI:** 10.1186/s13075-023-02999-8

**Published:** 2023-02-09

**Authors:** Hong-Ci Lin, Hsu-Min Chang, Yao-Min Hung, Renin Chang, Hsin-Hua Chen, James Cheng-Chung Wei

**Affiliations:** 1 School of Medicine, Department of Medicine, Chung Shan Medical University, Taichung, Taiwan; 2 Department of Medical Education and Research, Kaohsiung Veterans General Hospital, Kaohsiung, Taiwan; 3 Department of Internal Medicine, Kaohsiung Municipal United Hospital, Kaohsiung, Taiwan; 4 College of Health and Nursing, Meiho University, Pingtung, Taiwan; 5 Department of Emergency Medicine, Kaohsiung Veterans General Hospital, Kaohsiung, Taiwan; 6 Division of Allergy, Immunology and Rheumatology, Taichung Veterans General Hospital, Taichung, Taiwan; 7 Institute of Biomedical Science and Rong Hsing Research Centre for Translational Medicine, Chung Hsing University, Taichung, Taiwan; 8 Department of Industrial Engineering and Enterprise Information, Tunghai University, Taichung, Taiwan; 9 Division of Allergy, Immunology and Rheumatology, Chung Shan Medical University Hospital, Taichung, Taiwan; 10 Institute of Medicine, Chung Shan Medical University Hospital, Taichung, Taiwan; 11 Graduate Institute of Integrated Medicine, China Medical University, Taichung, Taiwan

**Keywords:** Hashimoto’s thyroiditis, Systemic lupus erythematosus, Retrospective cohort study

## Abstract

**Background:**

Previous studies have shown systemic lupus erythematosus (SLE) patients had a significantly higher prevalence of thyroid diseases and hypothyroidism than matched controls, and some case reports showed SLE may occur after Hashimoto’s thyroiditis (HT).

**Objective:**

This study aimed to investigate the subsequent risk of SLE in patients with HT.

**Methods:**

In this retrospective cohort study done by the Taiwan National Health Insurance Research Database, the HT group (exposure group) and the non-HT group (comparator group) were propensity score matched at a ratio of 1:2 by demographic data, comorbidities, medications, and the index date. We used Cox proportional hazards models to estimate hazard ratios (HRs) and 95% confidence intervals (CIs). Several sensitivity analyses were done for cross-validation of our findings.

**Results:**

We identified 15,512 HT patients and matched 31,024 individuals. The incidence rate ratio of SLE was 3.58 (95% CI, 2.43–5.28; *p* < 0.01). Several sensitivity analyses show adjusted hazard ratio (aHR) (CIs) of 4.35 (3.28–5.76), 4.39 (3.31–5.82), 5.11 (3.75–6.98), and 4.70 (3.46–6.38), consistent with the results of the main model.

**Conclusion:**

Our study showed an increased risk of SLE in the HT group after adjustment for baseline characteristics, comorbidities, and medical confounders compared with the reference group.

## Introduction

Systemic lupus erythematosus (SLE) is a systematic autoimmune disease affecting almost every organ, in which the immune system attacks tissues and cells leading to inflammation and damage [1]. Previous studies have shown that both the loss of B cell self-tolerance due to genetic factor and Th1 lymphocyte response are necessary for the development of SLE [2–4]. However, some studies indicated that genetic susceptibility alone is not sufficient to account for all SLE patients, with only 24% concordance within monozygotic twins with SLE, which is much lower than previous estimate [5]. Another study revealed that environmental exposures are more related to SLE. In this study, a higher concordance of autoimmune disease, including SLE, was presented among monozygotic twins than among dizygotic twins, which was explained as environment factors that monozygotic twins usually share are more similar than dizygotic do [6], rather than just considered as a genetic similarity. The environment factors for SLE include but not limited to ultraviolet radiation, infection, and hormone [7]. Besides, having a history of other autoimmune diseases is also a risk factor for another one, according to some studies about multiple autoimmune syndrome (MAS) [8, 9].

Studies also revealed that T_3_ and T_4_ act as modulators in the immune system [10, 11]. Thyroid hormone not only stimulates T cells, B cells [12, 13], neutrophils, and macrophage chemotaxis [14, 15] but also enhances the generation of ROS and IL-18 [16]. Despite that abundant studies about T_3_ and T_4_ alternating the immune status, we now only noticed that SLE patients are more likely to develop autoimmune thyroiditis or even hypothyroidism in some cohort study [2, 17], and there is no definite evidence that make sure whether hypothyroidism will cause SLE so far in spite of two case reports about two young girls and two women respectively evolving SLE after being diagnosed as hypothyroidism and Hashimoto’s thyroiditis (HT) [18, 19].

Based on the concept mentioned above, we hypothesized that a history of HT increases the risk of subsequent SLE, and, as the epidemiological correlation between HT and SLE has remained unclear, we designed a retrospective cohort study to investigate this issue.

## Materials and methods

### Method

#### Study design

In this national wide, retrospective cohort study with propensity score matched (PSM), the data was extracted from the National Health Insurance Research Database (NHIRD), which is a database constructed by 95% of residents in Taiwan since 1995 through the National health care insurance (NHI) system. Disease profiles are based on the International Classification of Diseases, 9th Revision, and Clinical Modification (ICD-9-CM) systems; the NHIRD provides information including demographics, outpatient visits, and hospitalizations with dates, prescriptions codes, diagnostic codes, laboratory tests and interventional procedure codes, and medical costs. De-identification was done for the protection of personal privacy.

We also extract the major illness registry data, also called the catastrophic illness registry, which is a certification for patients who were diagnosed with some severe, chronic, or fatal disease including cancer, diabetes mellitus, major injury, and SLE. The certification provides a discount on admission and medical charge. The Longitudinal Health Insurance Database 2000 (LHID 2000) was also used in the comparator group without HT of this study; LHID 2000 has collected a population of 1 million which was randomly sampled from the beneficiaries’ registration files within the year 2000. The representative of gender and age distribution has been statistically confirmed between the LHID 2000 and the origin NHIRD data.

#### Study population and propensity score match

We identified our population as patients who have had at least three outpatient visits or one hospital admission for autoimmune thyroiditis (Hashimoto’s thyroiditis) (ICD9 code: 245.2) between 2003 and 2012. The index date for the corresponding matching was defined as the date of the first diagnosis of autoimmune thyroiditis, outpatient, or admission.

We also constructed a comparator group without HT sampled from the LHID 2000 data, which includes patients ever visited outpatient departments between 2005 and 2012. The index date of the comparator group without HT was defined as the first visit to the outpatient department each year. Those who ever had at least one outpatient visit plus one hospitalization under the diagnosis of disorders of the thyroid gland (ICD9 codes 240–246: 240 for simple and unspecified goiter, 241 for nontoxic nodular goiter, 242 for thyrotoxicosis with or without goiter, 243 for congenital hypothyroidism, 244 for acquired hypothyroidism, 245 for thyroiditis, 246 for other disorders of the thyroid) between 1997 and 2013 were excluded.

We excluded the data of index date which were unmatched among the study and comparator group without HT (2003–2004), SLE (ICD9 code: 710.0) diagnosed before the index date and death during follow-up.

Overall, we extracted a total population of 17,978 cases with HT between the years 2005 and 2012 as our study group; and the comparator group without HT contained 783,345 patients without any disorders of the thyroid gland. To reduce the confounding bias, propensity score matching (PSM) was used, which was estimated by logistic regression modeling. Predictors involved index date, gender, and selected co-morbidities. The 1:2 matched comparator shares the same propensity score as the exposure group.

#### Outcomes and comorbidities

The primary outcome of the study was SLE occurrence, which was defined as patients who were diagnosed with SLE (ICD9 code: 710.0) and were identified as having “major illness” according to the NHI document for ensuring only correctly diagnosed patients were included. The follow-up started on the respective index date for different individuals until SLE was diagnosed or withdrawn from NHI due to any cause such as death, leaving, loss of data, or end of the study (December 2013), whichever occurred first. Relevant data of background variation including gender, age, urbanization, low income, length of hospital stays, times of outpatient department visits, medication control, and co-morbidities were also extracted and listed in Table 1.

**Table 1:** Baseline characteristics among the Hashimoto’s thyroiditis group and non-Hashimoto’s thyroiditis group

	Before PSM (1:20 age matching)				1:2 PSM			
	Non-Hashimoto’s thyroiditis, *n* = 315,020	Hashimoto’s thyroiditis, *n* = 15,751	*p* value	ASD	Non-Hashimoto’s thyroiditis, *n* = 31,024	Hashimoto’s thyroiditis, *n* = 15,512	*p* value	ASD
**Before, any time**								
Hyperthyroidism, hypothyroidism			< 0.001				< 0.001	
No hyperthyroidism and no hypothyroidism	314,531 (99.8)	6044 (38.4)			30,969 (99.8)	5910 (38.1)		
Hyperthyroidism only	294 (0.1)	2392 (15.2)			36 (0.1)	2367 (15.3)		
Hypothyroidism only	182 (0.1)	5604 (35.6)			18 (0.1)	5549 (35.8)		
Hyperthyroidism and hypothyroidism	13 (0.004)	1711 (10.9)			1 (0.003)	1686 (10.9)		
**Sex**			1.000	0.000			1.000	0.000
Female	271,700 (86.2)	13,585 (86.2)			26,750 (86.2)	13,375 (86.2)		
Male	43,320 (13.8)	2166 (13.8)			4274 (13.8)	2137 (13.8)		
**Age**	43.4 ± 16.0	43.4 ± 16.0	1.000	0.000	43.4 ± 16.0	43.4 ± 16.0	0.951	0.001
**Urbanization**			< 0.001	0.240			0.758	0.001
Urban	100,057 (31.8)	6638 (42.1)			13,003 (41.9)	6535 (42.1)		
Suburban	151,327 (48.0)	6944 (44.1)			13,798 (44.5)	6844 (44.1)		
Rural	63,636 (20.2)	2169 (13.8)			4223 (13.6)	2133 (13.8)		
**Low income**^c^	159,152 (50.5)	7078 (44.9)	< 0.001	0.112	13,957 (45.0)	6994 (45.1)	0.838	0.002
**3 months before the index date**								
Times of visiting the outpatient department	2.8 ± 4.1	6.6 ± 5.6	< 0.001		3.2 ± 4.4	6.5 ± 5.6	< 0.001	
Number of patients visited the outpatient department	182,102 (57.8)	14,898 (94.6)	< 0.001		19,243 (62)	14,661 (94.5)	< 0.001	
Length of hospital stays	0.2 ± 2.2	0.4 ± 3.1	< 0.001		0.3 ± 2.7	0.4 ± 3.0	0.002	
0 days	308,464 (97.9)	15,034 (95.4)	< 0.001		30,056 (96.9)	14,826 (95.6)	< 0.001	
1–6 days	3954 (1.3)	439 (2.8)			574 (1.9)	427 (2.8)		
≥ 7 days	2602 (0.8)	278 (1.8)			394 (1.3)	259 (1.7)		
**Follow up for 3 months after the index date**								
Times of visiting the outpatient department	5.2 ± 4.3	8.1 ± 5.5	< 0.001		5.5 ± 4.6	8.1 ± 5.5	< 0.001	
Number of patients visited the outpatient department	315,020 (100)	15,748 (100)	< 0.001		31,024 (100)	15,509 (100)	0.014	
Length of hospital stays	0.3 ± 2.8	0.7 ± 3.9	< 0.001		0.4 ± 3.1	0.6 ± 3.7	< 0.001	
0 days	304,835 (96.8)	14,602 (92.7)	< 0.001		29,976 (96.6)	14,456 (93.2)	< 0.001	
1–6 days	6269 (2.0)	676 (4.3)			636 (2.1)	632 (4.1)		
≥ 7 days	3916 (1.2)	473 (3.0)			412 (1.3)	424 (2.7)		
**Follow up for 6 months after the index date**								
Times of visiting the outpatient department	8.9 ± 7.8	14.4 ± 9.8	< 0.001		9.6 ± 8.2	14.3 ± 9.6	< 0.001	
Number of patients visited the outpatient department	315,020 (100)	15,749 (100)	< 0.001		31,024 (100)	15,510 (100)	0.045	
Length of hospital stays^a^	0.6 ± 4.9	1.0 ± 5.9	< 0.001		0.7 ± 5.3	0.9 ± 5.6	< 0.001	
0 days	298,484 (94.8)	14,170 (90.0)	< 0.001		29,253 (94.3)	14,029 (90.4)	< 0.001	
1–6 days	9996 (3.2)	933 (5.9)			1058 (3.4)	892 (5.8)		
≥ 7 days	6540 (2.1)	648 (4.1)			713 (2.3)	591 (3.8)		
**Co-morbidity**								
**1 previous year before the index date: outpatient department visits × 3, admission × 1**								
RA	819 (0.3)	167 (1.1)	< 0.001	0.099	203 (0.7)	134 (0.9)	0.012	0.024
SS	459 (0.1)	355 (2.3)	< 0.001	0.195	262 (0.8)	151 (1.0)	0.162	0.014
SSc	24 (0.01)	8 (0.1)	< 0.001	0.025	8 (0.03)	5 (0.03)	0.695	0.004
Vasculitis	33 (0.01)	17 (0.1)	< 0.001	0.040	17 (0.1)	8 (0.1)	0.888	0.001
Hypertension	34,921 (11.1)	2093 (13.3)	< 0.001	0.067	4294 (13.8)	2060 (13.3)	0.097	0.016
Diabetes mellitus	16,681 (5.3)	1090 (6.9)	< 0.001	0.068	2205 (7.1)	1073 (6.9)	0.450	0.007
Hyperlipidemia	13,795 (4.4)	1402 (8.9)	< 0.001	0.182	2795 (9.0)	1369 (8.8)	0.513	0.006
Coronary artery disease	7348 (2.3)	603 (3.8)	< 0.001	0.087	1110 (3.6)	586 (3.8)	0.278	0.011
Osteoporosis	2771 (0.9)	248 (1.6)	< 0.001	0.063	418 (1.3)	241 (1.6)	0.076	0.017
Cerebral vascular accident	4937 (1.6)	306 (1.9)	< 0.001	0.029	524 (1.7)	296 (1.9)	0.090	0.016
COPD/asthma	6898 (2.2)	541 (3.4)	< 0.001	0.075	1076 (3.5)	527 (3.4)	0.693	0.004
Chronic kidney disease	1632 (0.5)	91 (0.6)	0.310	0.008	147 (0.5)	91 (0.6)	0.108	0.016
Chronic liver diseases	6248 (2.0)	633 (4.0)	< 0.001	0.120	1250 (4.0)	601 (3.9)	0.421	0.008
Pancreatitis	286 (0.1)	27 (0.2)	0.001	0.022	42 (0.1)	24 (0.2)	0.601	0.005
Affective psychosis	1421 (0.5)	259 (1.6)	< 0.001	0.117	508 (1.6)	239 (1.5)	0.434	0.008
Ankylosing spondylitis	185 (0.1)	43 (0.3)	< 0.001	0.053	62 (0.2)	33 (0.2)	0.771	0.003
IBD	254 (0.1)	18 (0.1)	0.151	0.011	32 (0.1)	18 (0.1)	0.689	0.004
HIV infection	43 (0.01)	1 (0.01)	0.438	0.007	3 (0.01)	1 (0.01)	0.724	0.004
AIHA	3 (0.001)	5 (0.03)	< 0.001	0.024	1 (0.003)	1 (0.01)	0.617	0.005
ITP	36 (0.01)	15 (0.1)	< 0.001	0.036	19 (0.1)	9 (0.1)	0.894	0.001
**Hashimoto’s thyroiditis treatment at baseline**^b^								
No drug admiration		4139 (26.3)				4115 (26.5)		
Anti-thyroid medication (carbimazole, propylthiouracil, methimazole)/eltroxin only		8564 (54.4)				8538 (55.0)		
HCQ/corticosteroid+/− anti-thyroid medication/eltroxin		3048 (19.4)				2859 (18.4)		

^a^Length of hospital stay was identified within 2 years before the index date

^b^Hashimoto’s thyroiditis treatment was identified within 6 months after diagnosis with Hashimoto’s thyroiditis

^c^Insured income lower than median income (21,000 New Taiwan dollars/month)

To minimize surveillance bias, patients who were diagnosed with SLE during the period of 2 years before the index date were excluded. Besides, due to the chronic and latent nature of SLE, patients who were diagnosed with SLE whose follow-up time was less than 3 months and 6 months were excluded, respectively, in 2 scenarios, which were conducted to increase the accuracy, and in the background variations, groups of 3 months before the index date and 3 and 6 months after the index date were also corrected with length of hospital stays and times of outpatient department visits (Table 1).

Comorbidities were captured by tracing all the ambulatory care and admission records in the NHI database within 1 previous year of the index date and have had at least three outpatient visits or one hospital admission. We analyzed autoimmune disorders including rheumatoid arthritis (RA, ICD9 code: 714.0), Sjögren’s syndrome (SS, ICD9 code: 710.2), systemic sclerosis (SSc, ICD9 code: 710.1), and vasculitis (ICD9 code: 433.0) that not rarely occur with SLE. Other common comorbidities such as hypertension (ICD9 codes: 401–405), diabetes mellitus (ICD9 code: 250), hyperlipidemia (ICD9 codes: 272.0–272.4), coronary artery disease (ICD9 codes: 410–414), osteoporosis (ICD9 code: 733), cerebral vascular accident (ICD9 codes: 430–438), chronic obstructive pulmonary disease (COPD)/asthma (ICD9 codes: 490–496), chronic kidney disease (CKD, ICD9 code: 585), chronic liver diseases (ICD9 codes: 571, 573), pancreatitis (ICD9 codes: 577.0, 577.1), affective psychosis (ICD9 code: 296), ankylosing spondylitis (ICD9 code: 720.0), inflammatory bowel disease (ICD9 codes: 555–556), HIV infection (ICD9 codes: 042–044, V08), autoimmune hemolytic anemia (AIHA) (ICD9 code: 283.0), and idiopathic thrombocytopenic purpura (ITP) (ICD9 code: 287.3) were also included in the study. Baseline treatment of HT including (1) no drug admiration, (2) anti-thyroid medication (carbimazole, propylthiouracil, methimazole)/eltroxin only, and (3) HCQ/corticosteroid+/− anti-thyroid medication/eltroxin was also analyzed, and all treatments were given within 6 months after diagnosis. Besides, hyperthyroidism (ICD9 code: 242) and hypothyroidism (ICD9 codes: 243, 244) diagnosed before the index date were separately analyzed by multivariable statistical analysis, which was listed in Tables 5, 6, and 7.

#### Statistical analysis

To compare and increase the similarities between our exposure group of HT and the comparator group without HT, the chi-square (*χ*^2^) tests and the two-tailed *T* test was used for the baseline demographic characteristics such as gender, age, urbanization, income level, admission duration, and comorbidities. Time-to-event analysis was conducted based on the index date defined as the fixed time point (January 2005) for every participation. All participants were followed up from their respective index date until the occurrence of SLE, until withdrawal, or until the end of 2013, whichever occurred first.

We also constructed a multivariable Cox proportional hazard model to estimate the hazard ratios (HRs) and 95% confidence intervals (CIs) for the SLE incidence. In the 1:20 age- and gender-matched population, 3 models were conducted. The first would be the model of HT alone, which also analyzed other thyroid disorders. For the second model, hyperthyroid and hypothyroid disorders were excluded. Model 3 contains HT with demographic variables, medical utilization, and comorbidities, and for the 1:2 PSM population, models A and B were constructed by controlling variables such as HT, hyperthyroid, and hypothyroid disorders. All the data and statistics were processed and analyzed by the Statistics Analysis System (SAS) software version 9.3 (SAS Institute, Inc., Cary, NC), and a *p*-value less than 0.05 was considered to indicate statistical significance.

#### Sensitivity analysis

To test the reliability of our study results, we established 4 sensitivity analysis scenarios including SLE medication treatment and the exclusion of autoimmune thyroiditis with other autoimmune diseases. The SLE treatment was identified as systemic corticosteroids or disease-modifying anti-rheumatic drugs (DMARDs) (including hydroxychloroquine (HCQ) or azathioprine) within 6 months after the first diagnosis of SLE. In scenarios 1–3, adjusted hazard ratio (aHR) was analyzed based on a different definition of SLE event. The main finding without medication treatment analysis would be scenario 1, systemic corticosteroids or DMARDs treatment were brought into scenario 2, and systemic corticosteroids were excluded in scenario 3. Scenario 4 modified the exclusion criteria and exclusion of the patients with rheumatic arthritis (RA), Sjögren’s syndrome (SS), systematic sclerosis (SSc), vasculitis, ankylosing spondylitis (AS), and inflammatory bowel disease (IBD) at baseline; hence, the autoimmune thyroiditis accompanied with other autoimmune diseases could be ruled out. The sensitivity analysis scenarios were listed in Tables 8, 9, and 10.

## Results

After exclusion and 1:20 age match, we identified 15,751 patients among 25,018 HT patients as our study group; 315,020 patients were extracted for the comparator group without HT. Furthermore, the 1:2 PSM filtered out 15,512 cases for the study group with 31,024 cases for the comparator group without HT. The baseline demographic characteristics, medical utilizations, and comorbidities of both groups were listed in Table 1. There was a significant higher proportion of most listed comorbidities (*p* < 0.001) except for CKD, IBD, and HIV infection in the HT group. As for the baseline medical treatment, over half of the cases (54.4%) in this study were under anti-thyroid medication only, such as carbimazole, propylthiouracil, methimazole, and eltroxin; about 26.3% of cases without medication control; and 19.3% patients were taking HCQ or corticosteroids. Also, Table 1 contained the baseline proportion of thyroid function disorders, which leads to our further multivariable Cox regression analysis.

**Fig. 1:**
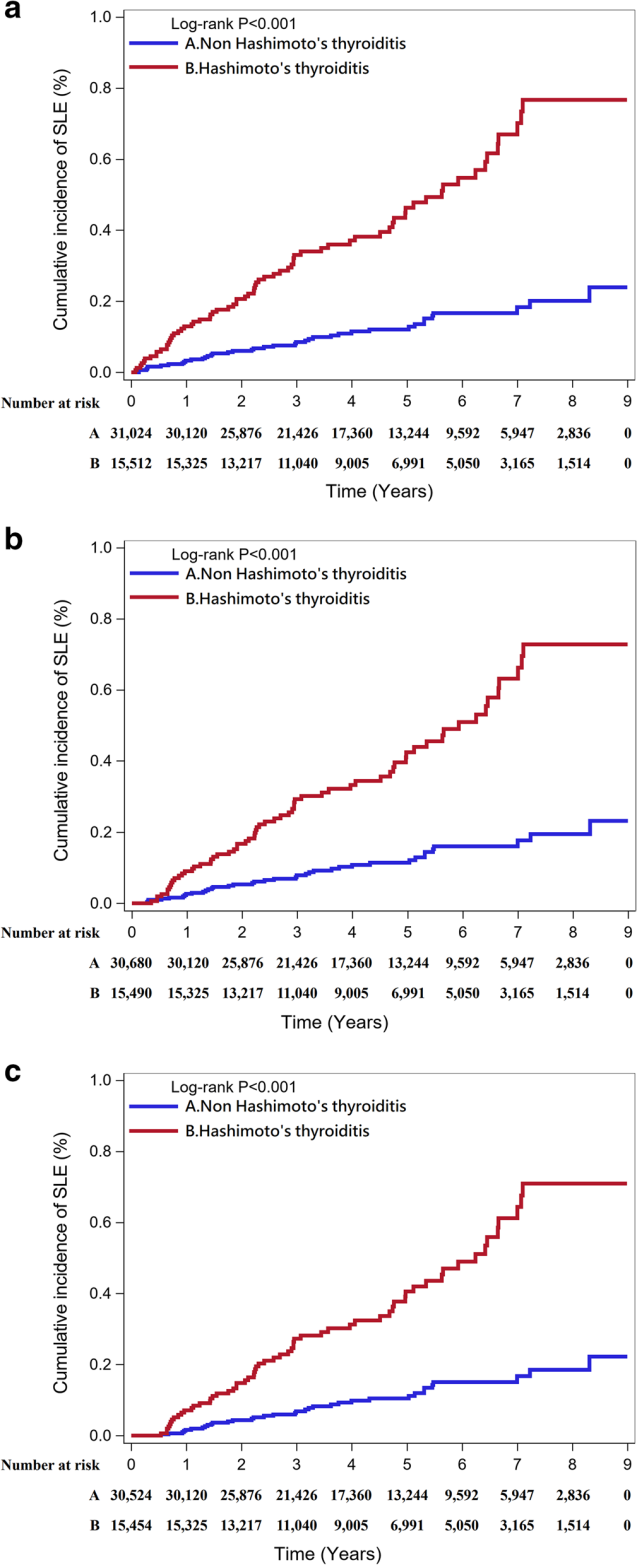
**a** The cumulative probability of SLE in non-HT and HT patients after PSM 1:2. **b** The cumulative probability of SLE in non-HT and HT patients after PSM 1:2. Follow-up duration of samples ≧ 3 months. **c** The cumulative probability of SLE in non-HT and HT patients after PSM 1:2. Follow-up duration of samples ≧ 6 months

**Fig. 2:**
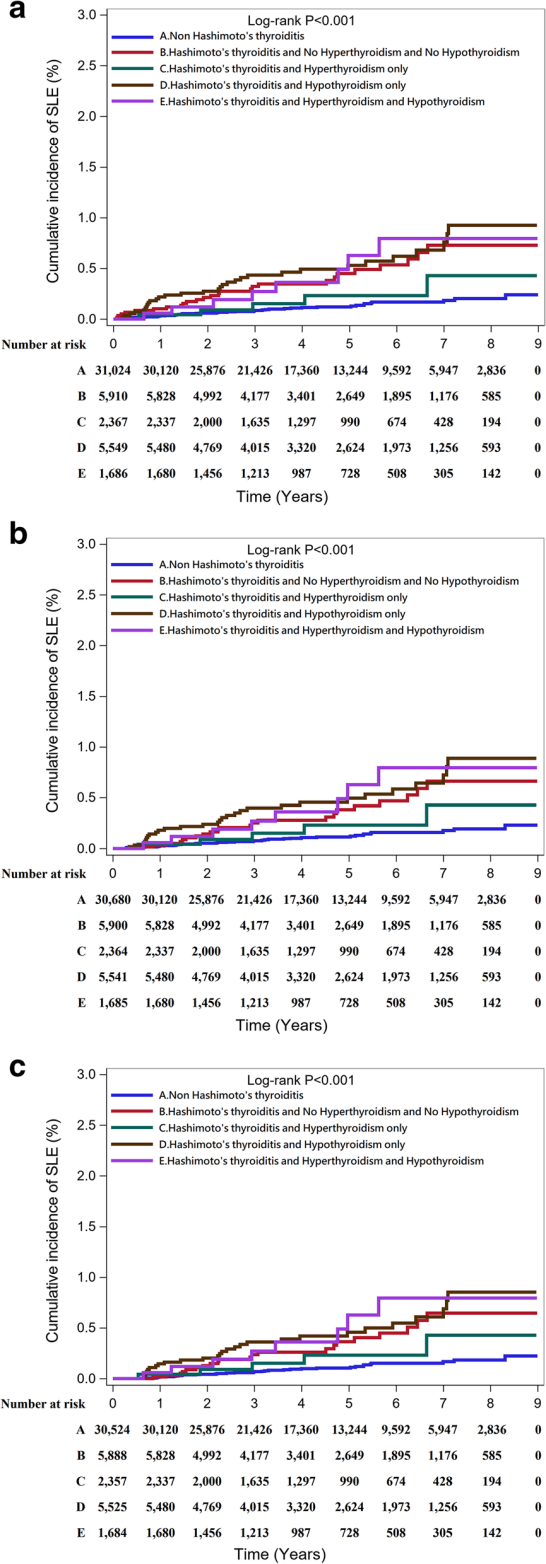
**a** The cumulative probability of SLE in non-HT and HT patients with hyperthyroidism or hypothyroidism after PSM 1:2. **b** The cumulative probability of SLE in non-HT and HT patients with hyperthyroidism or hypothyroidism after PSM 1:2. Follow-up duration of samples ≧ 3 months. **c** The cumulative probability of SLE in non-HT and HT patients with hyperthyroidism or hypothyroidism after PSM 1:2. Follow-up duration of samples ≧ 6 months

Tables 2, 3, and 4 present the time-to-event analysis of the SLE incidence rate, including sensitivity analysis scenarios listed in Tables 8, 9, and 10, including data before and after PSM. Before PSM, the incidence rate ratio was similar in scenarios 1–3 (6.83, 95% CI: 5.35–8.72; 6.86, 95% CI: 5.37–8.77; 7.35 95% CI: 5.60–9.63, respectively) and slightly lower in scenario 4 (5.53, 95% CI: 4.16–7.35) which excluded other autoimmune disorders that might have a symptom of thyroiditis. However, after 1:2 PSM, the incidence rate ratio in scenario 4 became slightly higher than in scenarios 1–2 and still lower than in scenario 3. These results were also noted even though we set more barriers on following time, which are presented in Tables 3 and 4. Overall, patients with HT presented a significant increasing risk of SLE in all 4 scenarios (*p*, long rank *p* < 0.001). The cumulative probability of SLE incidence after PSM 1:2 was presented via Kaplan-Meier curves, and they were analyzed with the shortest follow-up period of 3 months and 6 months (Fig. 1a–c). In Fig. 2a–c, the factors of hyperthyroidism and hypothyroidism were discussed also. Their data were shown in Table 11.

**Table 2:** Incidence rate. No restriction for follow-up duration

Variable	Total	Event (%)	Total person-years	Incidence rate (/10^5^ years)	IRR (95%CI)	*p* value	Log-rank *p*	Proportional hazards assumption
**Before PSM (1:20 age, sex matching)**								
**Scenario 1**								
Non-Hashimoto’s thyroiditis	315,020	258 (0.08)	1,512,453	17.06	Ref.		< 0.001	0.849
Hashimoto’s thyroiditis	15,751	86 (0.55)	73,793	116.54	6.83 (5.35–8.72)	< 0.001		
**Scenario 2**								
Non-Hashimoto’s thyroiditis	315,020	254 (0.08)	1,512,465	16.79	Ref.		< 0.001	0.790
Hashimoto’s thyroiditis	15,751	85 (0.54)	73,795	115.18	6.86 (5.37–8.77)	< 0.001		
**Scenario 3**								
Non-Hashimoto’s thyroiditis	315,020	198 (0.06)	1,512,698	13.09	Ref.		< 0.001	0.249
Hashimoto’s thyroiditis	15,751	71 (0.45)	73,835	96.16	7.35 (5.60–9.63)	< 0.001		
**Scenario 4**								
Non-Hashimoto’s thyroiditis	313,366	228 (0.07)	1,504,643	15.15	Ref.		< 0.001	0.253
Hashimoto’s thyroiditis	15,201	60 (0.39)	71,563	83.84	5.53 (4.16–7.35)	< 0.001		
**1:2 PSM**								
**Scenario 1**								
Non-Hashimoto’s thyroiditis	31,024	40 (0.13)	149,219	26.81	Ref.		< 0.001	0.999
Hashimoto’s thyroiditis	15,512	70 (0.45)	72,894	96.03	3.58 (2.43–5.28)	< 0.001		
**Scenario 2**								
Non-Hashimoto’s thyroiditis	31,024	38 (0.12)	149,227	25.46	Ref.		< 0.001	0.922
Hashimoto’s thyroiditis	15,512	70 (0.45)	72,894	96.03	3.77 (2.54–5.60)	< 0.001		
**Scenario 3**								
Non-Hashimoto’s thyroiditis	31,024	23 (0.07)	149,267	15.41	Ref.		< 0.001	0.194
Hashimoto’s thyroiditis	15,512	59 (0.38)	72,926	80.90	5.25 (3.24–8.50)	< 0.001		
**Scenario 4**								
Non-Hashimoto’s thyroiditis	30,501	28 (0.09)	146,799	19.07	Ref.		< 0.001	0.832
Hashimoto’s thyroiditis	15,180	58 (0.38)	71,496	81.12	4.25 (2.71–6.68)	< 0.001

**Table 3:** Incidence rate. Follow-up duration of samples ≧ 3 months

Variable	Total	Event (%)	Total person-years	Incidence rate (/10^5^ years)	IRR (95%CI)	*p* value	Log-rank *p*	Proportional hazards assumption
**Before PSM (1:20 age, sex matching)**								
**Scenario 1**								
Non-Hashimoto’s thyroiditis	310,704	241 (0.08)	1,512,207	15.94	Ref.		< 0.001	0.478
Hashimoto’s thyroiditis	15,725	76 (0.48)	73,789	103.00	6.46 (4.99–8.36)	< 0.001		
Scenario 2								
Non-Hashimoto’s thyroiditis	310,704	237 (0.08)	1,512,219	15.67	Ref.		< 0.001	0.510
Hashimoto’s thyroiditis	15,726	76 (0.48)	73,791	102.99	6.57 (5.08–8.51)	< 0.001		
Scenario 3								
Non-Hashimoto’s thyroiditis	310,709	186 (0.06)	1,512,452	12.30	Ref.		< 0.001	0.202
Hashimoto’s thyroiditis	15,730	66 (0.42)	73,832	89.39	7.27 (5.49–9.63)	< 0.001		
Scenario 4								
Non-Hashimoto’s thyroiditis	309,068	214 (0.07)	1,504,399	14.22	Ref.		< 0.001	0.125
Hashimoto’s thyroiditis	15,180	54 (0.36)	71,560	75.46	5.30 (3.94–7.15)	< 0.001		
**1:2 PSM**								
**Scenario 1**								
Non-Hashimoto’s thyroiditis	30,680	38 (0.12)	149,198	25.47	Ref.		< 0.001	0.810
Hashimoto’s thyroiditis	15,490	64 (0.41)	72,891	87.80	3.45 (2.31–5.15)	< 0.001		
**Scenario 2**								
Non-Hashimoto’s thyroiditis	30,680	36 (0.12)	149,206	24.13	Ref.		< 0.001	0.910
Hashimoto’s thyroiditis	15,490	64 (0.41)	72,891	87.80	3.64 (2.42–5.47)	< 0.001		
**Scenario 3**								
Non-Hashimoto’s thyroiditis	30,680	21 (0.07)	149,246	14.07	Ref.		< 0.001	0.246
Hashimoto’s thyroiditis	15,493	56 (0.36)	72,923	76.79	5.46 (3.31–9.01)	< 0.001		
**Scenario 4**								
Non-Hashimoto’s thyroiditis	30,159	27 (0.09)	146,778	18.40	Ref.		< 0.001	0.603
Hashimoto’s thyroiditis	15,160	53 (0.35)	71,493	74.13	4.03 (2.54–6.41)	< 0.001

**Table 4:** Incidence rate. Follow-up duration of samples ≧ 6 months

Variable	Total	Event (%)	Total person-years	Incidence rate (/10^5^ years)	IRR (95%CI)	*p* value	Log-rank *p*	Proportional hazards assumption
**Before PSM (1:20 age, sex matching)**								
**Scenario 1**								
Non-Hashimoto’s thyroiditis	309,065	218 (0.07)	1,511,589	14.42	Ref.		< 0.001	0.581
Hashimoto’s thyroiditis	15,685	70 (0.45)	73,774	94.88	6.58 (5.03–8.61)	< 0.001		
**Scenario 2**								
Non-Hashimoto’s thyroiditis	309,065	214 (0.07)	1,511,601	14.16	Ref.		< 0.001	0.631
Hashimoto’s thyroiditis	15,686	70 (0.45)	73,776	94.88	6.70 (5.12–8.78)	< 0.001		
**Scenario 3**								
Non-Hashimoto’s thyroiditis	309,075	168 (0.05)	1,511,836	11.11	Ref.		< 0.001	0.259
Hashimoto’s thyroiditis	15,691	61 (0.39)	73,817	82.64	7.44 (5.55–9.97)	< 0.001		
**Scenario 4**								
Non-Hashimoto’s thyroiditis	307,441	195 (0.06)	1,503,786	12.97	Ref.		< 0.001	0.259
Hashimoto’s thyroiditis	15,147	52 (0.34)	71,547	72.68	5.60 (4.13–7.61)	< 0.001		
**1:2 PSM**								
**Scenario 1**								
Non-Hashimoto’s thyroiditis	30,524	35 (0.11)	149,139	23.47	Ref.		< 0.001	0.999
Hashimoto’s thyroiditis	15,454	61 (0.39)	72,877	83.70	3.57 (2.35–5.40)	< 0.001		
**Scenario 2**								
Non-Hashimoto’s thyroiditis	30,524	33 (0.11)	149,147	22.13	Ref.		< 0.001	0.871
Hashimoto’s thyroiditis	15,454	61 (0.39)	72,877	83.70	3.78 (2.48–5.78)	< 0.001		
**Scenario 3**								
Non-Hashimoto’s thyroiditis	30,524	18 (0.06)	149,187	12.07	Ref.		< 0.001	0.472
Hashimoto’s thyroiditis	15,457	53 (0.34)	72,909	72.69	6.02 (3.53–10.28)	< 0.001		
**Scenario 4**								
Non-Hashimoto’s thyroiditis	30,004	25 (0.08)	146,719	17.04	Ref.		< 0.001	0.844
Hashimoto’s thyroiditis	15,129	52 (0.34)	71,481	72.75	4.27 (2.65–6.88)	< 0.001

Tables 5, 6, and 7 show the results of univariable and multivariable Cox regression analyses. The adjusted hazard ratio in HT exposure alone (model 1) was 6.79 (95% CI: 5.32–8.66), which indicates that the increased risk of SLE in the HT exposure and other thyroid disorders was also analyzed, and shows that patients with either HT, hyperthyroidism, or hypothyroidism were all supposed to have an increased SLE incident risk. The aHR of model 2a, with demographic adjustment including sex, age, urbanization, low income, length of hospital stays at baseline, and times of outpatient department visits, was 5.83 (95% CI: 4.50–7.56) and showed increasing risk on long hospital stay patients. Model 3a shows that the aHR after adjustment of demographic variables, medical utilization, and comorbidities at baseline was 4.35 (95% CI: 3.28–5.76). Autoimmune diseases listed in the table including RA, SS, SSc, vasculitis, AIHA, and ITP as comorbidities also increased the incidence rate of SLE compared with the comparator group without HT. Models 2b and 3b included the variations of HT, hyperthyroidism, and hypothyroidism; these models still reveal similar results. HT, hyperthyroidism, and hypothyroidism were all supposed to increase the SLE incident risk, and the HT only group shows the highest aHR of 6.52 (4.55–9.34) in model 2b and second highest aHR of 4.53 (3.08–6.66) in model 3b; compared to HT combined with hyperthyroidism, those who have combined HT and hypothyroidism were more likely to develop SLE. Demographic variables were similar to the previous description. As for the comorbidities analysis, a high hazard ratio of SLE was also found in other autoimmune diseases such as RA, SS, SSc, and vasculitis.

**Table 5:** Cox proportional hazard regressions for estimation of adjusted HRs

	1:20 age-matched and sex-matched population					1:2 PSM population	
	Model 1: Hashimoto’s thyroiditis exposure alone	Model 2a: Hashimoto’s thyroiditis exposure + demographic variables	Model 3a: model 2 + medical utilization and comorbidities at baseline	Model 2b: Hashimoto’s thyroiditis exposure + demographic variables	Model 3b: model 2 + medical utilization and comorbidities at baseline	Model A: conditional Cox model with Hashimoto’s thyroiditis exposure alone	Model B: conditional Cox model with Hashimoto’s thyroiditis exposure, hyperthyroidism, and hypothyroidism
**Hashimoto’s thyroiditis**	6.79 (5.32–8.66)	5.83 (4.50–7.56)	4.35 (3.28–5.76)			3.54 (2.40–5.22)	
**Hyperthyroidism, hypothyroidism**							
No Hashimoto’s thyroiditis	Ref.			Ref.	Ref.		Ref.
Hashimoto’s thyroiditis, no hyperthyroidism, and no hypothyroidism	7.46 (5.26–10.57)			6.52 (4.55–9.34)	4.53 (3.08–6.66)		3.47 (2.12–5.69)
Hashimoto’s thyroiditis and hyperthyroidism only	3.21 (1.43–7.21)			2.88 (1.27–6.50)	2.63 (1.16–5.96)		1.72 (0.68–4.35)
Hashimoto’s thyroiditis and hypothyroidism only	7.59 (5.34–10.81)			6.40 (4.45–9.21)	4.99 (3.41–7.32)		4.27 (2.67–6.83)
Hashimoto’s thyroiditis, hyperthyroidism, and hypothyroidism	6.59 (3.39–12.81)			5.29 (2.70–10.36)	3.67 (1.83–7.37)		3.77 (1.76–8.05)
**Sex—male**	0.19 (0.10–0.36)	0.18 (0.09–0.35)	0.20 (0.10–0.39)	0.18 (0.10–0.36)	0.20 (0.10–0.39)		
**Age**	1.00 (0.99–1.01)	1.00 (0.99–1.004)	0.99 (0.98–0.99)	1.00 (0.99–1.004)	0.99 (0.98–0.99)		
**Urbanization**							
Urban	Ref.		Ref.	Ref.	Ref.		
Suburban	0.91 (0.72–1.16)	0.98 (0.77–1.25)	0.96 (0.76–1.23)	0.99 (0.77–1.26)	0.97 (0.76–1.24)		
Rural	1.04 (0.78–1.40)	1.17 (0.87–1.57)	1.11 (0.83–1.49)	1.17 (0.87–1.57)	1.11 (0.83–1.50)		
**Low income (≤** m**edian 21,000 NTD/month)**	1.09 (0.88–1.35)	1.10 (0.89–1.36)	1.04 (0.84–1.29)	1.09 (0.88–1.35)	1.04 (0.84–1.29)		
**3 months before the index date**							
**Times of visiting the outpatient department**	1.06 (1.05–1.08)	1.04 (1.02–1.06)	1.01 (0.99–1.03)	1.04 (1.02–1.06)	1.01 (0.99–1.03)		
Length of hospital stays*							
0 days	Ref.	Ref.	Ref.	Ref.	Ref.		
1–6 days	2.41 (1.28–4.52)	1.71 (0.90–3.23)	1.39 (0.72–2.67)	1.74 (0.92–3.29)	1.43 (0.74–2.76)		
≥ 7 days	5.30 (2.90–9.66)	3.85 (2.08–7.13)	2.72 (1.42–5.20)	3.88 (2.09–7.18)	2.71 (1.41–5.19)		
**Co-morbidity**							
**1 previous year of the index date: OPD visits × 3, admission × 1**							
RA	22.12 (14.23–34.40)		3.82 (2.20–6.65)		3.74 (2.14–6.52)		
SS	50.39 (35.35–71.82)		11.40 (7.13–18.22)		11.29 (7.03–18.15)		
SSc	133.44 (49.86–357.09)		6.73 (2.24–20.18)		6.88 (2.26–20.95)		
Vasculitis	103.27 (42.71–249.70)		7.24 (2.66–19.69)		6.99 (2.55–19.16)		
Hypertension	1.31 (0.96–1.79)		1.46 (1.01–2.13)		1.46 (1.01–2.13)		
Diabetes mellitus	0.64 (0.35–1.16)		0.49 (0.26–0.93)		0.49 (0.26–0.94)		
Hyperlipidemia	1.02 (0.61–1.71)		0.79 (0.45–1.37)		0.77 (0.44–1.34)		
Coronary artery disease	1.05 (0.52–2.11)		0.67 (0.32–1.42)		0.68 (0.32–1.44)		
Osteoporosis	5.01 (2.99–8.40)		2.89 (1.66–5.05)		2.88 (1.65–5.03)		
Cerebral vascular accident	1.75 (0.87–3.53)		1.27 (0.60–2.69)		1.25 (0.59–2.65)		
COPD/asthma	2.22 (1.35–3.67)		1.44 (0.84–2.47)		1.44 (0.84–2.47)		
Chronic kidney disease	2.22 (0.71–6.91)		2.00 (0.63–6.38)		1.95 (0.61–6.23)		
Chronic liver diseases	3.23 (2.08–5.03)		2.28 (1.42–3.65)		2.27 (1.42–3.65)		
Pancreatitis	3.67 (0.52–26.11)		1.18 (0.16–8.91)		1.15 (0.15–8.77)		
Affective psychosis	2.37 (0.88–6.35)		1.27 (0.45–3.58)		1.29 (0.46–3.63)		
Ankylosing spondylitis	4.25 (0.60–30.28)		0.73 (0.08–6.36)		0.79 (0.09–6.74)		
IBD	0.00 (0.00–6.17E135)		0.00 (0.00–1.85E178)		0.00 (0.00–5.59E177)		
HIV infection	0.00 (0.00–2.35E139)		0.00 (0.00–9.99E999)		0.00 (0.00–9.99E999)		
AIHA	322.78 (45.35–2297.39)		133.96 (18.18–986.89)		127.00 (17.22–936.73)		
ITP	81.51 (30.42–218.43)		36.62 (13.18–101.76)		37.20 (13.32–103.90)

**Table 6:** Cox proportional hazard regressions for estimation of adjusted HRs. Follow-up duration of samples≧3 months

	1:20 age-matched and sex-matched population					1:2 PSM population	
	Model 1: Hashimoto’s thyroiditis exposure alone	Model 2a: Hashimoto’s thyroiditis exposure + demographic variables	Model 3a: model 2 + medical utilization and comorbidities at baseline	Model 2b: Hashimoto’s thyroiditis exposure + demographic variables	Model 3b: model 2 + medical utilization and comorbidities at baseline	Model A: conditional Cox model with Hashimoto’s thyroiditis exposure alone	Model B: conditional Cox model with Hashimoto’s thyroiditis exposure, hyperthyroidism, and hypothyroidism
**Hashimoto’s thyroiditis**	6.44 (4.97–8.33)	5.05 (3.84–6.65)	3.84 (2.84–5.19)			3.41 (2.28–5.10)	
**Hyperthyroidism, hypothyroidism**							
No Hashimoto’s thyroiditis	Ref.			Ref.	Ref.		Ref.
Hashimoto’s thyroiditis, no hyperthyroidism, and no hypothyroidism	6.89 (4.74–10.02)			5.36 (3.65–7.89)	3.81 (2.51–5.79)		3.10 (1.83–5.24)
Hashimoto’s thyroiditis and hyperthyroidism only	3.45 (1.54–7.76)			2.75 (1.21–6.23)	2.66 (1.17–6.05)		1.82 (0.71–4.61)
Hashimoto’s thyroiditis and hypothyroidism only	7.20 (4.96–10.47)			5.74 (3.91–8.43)	4.43 (2.94–6.67)		4.21 (2.60–6.82)
Hashimoto’s thyroiditis, hyperthyroidism, and hypothyroidism	6.29 (3.11–12.73)			4.67 (2.29–9.51)	3.43 (1.63–7.23)		3.97 (1.85–8.52)
**Sex—male**	0.18 (0.09–0.37)	0.17 (0.08–0.34)	0.19 (0.10–0.39)	0.17 (0.09–0.35)	0.19 (0.10–0.39)		
**Age**	1.00 (0.99–1.01)	0.99 (0.99–1.001)	0.99 (0.98–0.99)	0.99 (0.99–1.001)	0.99 (0.98–0.99)		
**Urbanization**							
Urban	Ref.	Ref.	Ref.	Ref.	Ref.		
Suburban	0.93 (0.72–1.20)	0.99 (0.77–1.27)	0.98 (0.76–1.26)	0.99 (0.77–1.27)	0.98 (0.76–1.27)		
Rural	1.09 (0.81–1.48)	1.19 (0.88–1.62)	1.16 (0.85–1.58)	1.19 (0.88–1.61)	1.16 (0.85–1.58)		
**Low income (≤** m**edian 21,000 NTD/month)**	1.08 (0.87–1.34)	1.05 (0.84–1.31)	1.02 (0.81–1.28)	1.04 (0.84–1.30)	1.02 (0.81–1.27)		
**3 months before the index date**							
**Times of visiting the outpatient department**	1.06 (1.05–1.08)	0.99 (0.97–1.02)	0.97 (0.95–1.000)	0.99 (0.97–1.02)	0.97 (0.95–1.001)		
Length of hospital stays*							
0 days	Ref.	Ref.	Ref.	Ref.	Ref.		
1–6 days	2.09 (1.04–4.22)	1.35 (0.66–2.74)	1.09 (0.52–2.30)	1.36 (0.67–2.78)	1.12 (0.53–2.37)		
≥ 7 days	5.34 (2.84–10.02)	2.30 (1.18–4.49)	2.08 (1.04–4.16)	2.30 (1.18–4.50)	2.07 (1.03–4.16)		
**3 months after the index date**							
**Times of visiting the outpatient department**	1.07 (1.06–1.08)	1.06 (1.03–1.08)	1.06 (1.04–1.09)	1.06 (1.03–1.08)	1.06 (1.04–1.09)		
Length of hospital stays*							
0 days	Ref.	Ref.	Ref.	Ref.	Ref.		
1–6 days	2.93 (1.77–4.85)	2.00 (1.20–3.33)	1.36 (0.78–2.36)	2.03 (1.22–3.39)	1.36 (0.78–2.36)		
≥7 days	10.17 (6.75–15.32)	5.99 (3.84–9.35)	3.77 (2.33–6.11)	6.06 (3.88–9.46)	3.80 (2.35–6.16)		
**Co-morbidity**							
**1 previous year of the index date: OPD visits × 3, admission × 1**							
RA	21.73 (13.67–34.55)		4.21 (2.36–7.52)		4.18 (2.33–7.48)		
SS	46.80 (31.94–68.59)		8.12 (4.86–13.58)		8.01 (4.77–13.46)		
SSc	109.05 (35.03–339.49)		3.28 (0.91–11.88)		3.46 (0.94–12.72)		
Vasculitis	89.68 (33.46–240.33)		6.77 (2.19–20.88)		6.48 (2.09–20.13)		
Hypertension	1.27 (0.92–1.76)		1.30 (0.88–1.94)		1.30 (0.87–1.93)		
Diabetes mellitus	0.63 (0.34–1.19)		0.48 (0.25–0.95)		0.49 (0.25–0.96)		
Hyperlipidemia	1.04 (0.61–1.78)		0.83 (0.47–1.48)		0.82 (0.46–1.45)		
Coronary artery disease	1.15 (0.57–2.31)		0.71 (0.33–1.52)		0.71 (0.33–1.53)		
Osteoporosis	5.08 (2.97–8.68)		2.75 (1.54–4.92)		2.74 (1.54–4.91)		
Cerebral vascular accident	1.93 (0.96–3.90)		1.19 (0.55–2.57)		1.17 (0.54–2.53)		
COPD/asthma	2.27 (1.35–3.81)		1.48 (0.85–2.56)		1.47 (0.85–2.55)		
Chronic kidney disease	1.64 (0.41–6.59)		1.42 (0.35–5.82)		1.40 (0.34–5.76)		
Chronic liver diseases	3.37 (2.14–5.29)		2.24 (1.37–3.65)		2.22 (1.36–3.62)		
Pancreatitis	4.04 (0.57–28.78)		0.78 (0.10–6.22)		0.78 (0.10–6.29)		
Affective psychosis	1.28 (0.32–5.15)		0.47 (0.10–2.16)		0.48 (0.11–2.18)		
Ankylosing spondylitis	4.63 (0.65–32.92)		0.76 (0.08–7.10)		0.81 (0.09–7.33)		
IBD	0.00 (0.00–1.97E141)		0.00 (0.00–4.81E157)		0.00 (0.00–3.17E156)		
HIV infection	0.00 (0.00–2.91E146)		0.00 (0.00–9.99E999)		0.00 (0.00–9.99E999)		
AIHA	408.92 (57.36–2915.47)		133.16 (17.51–1012.64)		125.86 (16.48–961.45)		
ITP	44.25 (11.02–177.67)		15.18 (3.63–63.38)		15.42 (3.68–64.63)

**Table 7:** Cox proportional hazard regressions for the estimation of adjusted HRs. Follow-up duration of samples ≧ 6 months

	1:20 age-matched and sex-matched population					1:2 PSM population	
	Model 1: Hashimoto’s thyroiditis exposure alone	Model 2a: Hashimoto’s thyroiditis exposure + demographic variables	Model 3a: model 2 + medical utilization and comorbidities at baseline	Model 2b: Hashimoto’s thyroiditis exposure + demographic variables	Model 3b: model 2 + medical utilization and comorbidities at baseline	Model A: conditional Cox model with Hashimoto’s thyroiditis exposure alone	Model B: conditional Cox model with Hashimoto’s thyroiditis exposure, hyperthyroidism, and hypothyroidism
**Hashimoto’s thyroiditis**	6.58 (5.02–8.61)	5.26 (3.95–7.01)	4.13 (3.02–5.65)			3.54 (2.33–5.36)	
**Hyperthyroidism, hypothyroidism**							
No Hashimoto’s thyroiditis	Ref.			Ref.	Ref.		Ref.
Hashimoto’s thyroiditis, no hyperthyroidism, and no hypothyroidism	6.91 (4.67–10.25)			5.58 (3.72–8.36)	4.10 (2.65–6.36)		3.22 (1.87–5.53)
Hashimoto’s thyroiditis and hyperthyroidism only	3.85 (1.71–8.65)			3.11 (1.37–7.05)	2.97 (1.30–6.75)		1.98 (0.78–5.05)
Hashimoto’s thyroiditis and hypothyroidism only	7.21 (4.86–10.68)			5.79 (3.86–8.68)	4.65 (3.03–7.14)		4.25 (2.57–7.03)
Hashimoto’s thyroiditis and hyperthyroidism and hypothyroidism	6.99 (3.45–14.15)			5.26 (2.57–10.76)	3.94 (1.87–8.33)		4.33 (2.01–9.32)
**Sex—male**	0.20 (0.10–0.41)	0.19 (0.10–0.39)	0.21 (0.11–0.43)	0.20 (0.10–0.40)	0.21 (0.11–0.43)		
**Age**	1.00 (0.99–1.01)	0.99 (0.99–1.001)	0.99 (0.98–0.99)	0.99 (0.99–1.001)	0.99 (0.98–0.99)		
**Urbanization**							
Urban	Ref.	Ref.	Ref.	Ref.	Ref.		
Suburban	0.98 (0.76–1.28)	1.05 (0.80–1.37)	1.03 (0.79–1.35)	1.05 (0.80–1.37)	1.04 (0.79–1.35)		
Rural	1.05 (0.76–1.45)	1.14 (0.82–1.58)	1.10 (0.79–1.53)	1.14 (0.82–1.58)	1.10 (0.79–1.53)		
**Low income (≤** m**edian 21,000 NTD/month)**	1.06 (0.84–1.34)	1.04 (0.82–1.31)	1.02 (0.80–1.29)	1.04 (0.82–1.31)	1.01 (0.80–1.28)		
**3 months before the index date**							
**Times of visiting the outpatient department**	1.06 (1.05–1.08)	0.99 (0.96–1.02)	0.97 (0.94–1.01)	0.99 (0.96–1.02)	0.97 (0.94–1.01)		
Length of hospital stays*							
0 days	Ref.	Ref.	Ref.	Ref.	Ref.		
1–6 days	1.72 (0.77–3.86)	1.09 (0.48–2.48)	0.93 (0.40–2.17)	1.11 (0.49–2.51)	0.95 (0.40–2.22)		
≥ 7 days	4.79 (2.37–9.67)	2.11 (1.01–4.41)	1.90 (0.88–4.09)	2.10 (1.004–4.41)	1.90 (0.88–4.08)		
**6 months after the index date**							
**Times of visiting the outpatient department**	1.04 (1.03–1.04)	1.03 (1.01–1.04)	1.03 (1.01–1.04)	1.03 (1.01–1.04)	1.03 (1.01–1.04)		
Length of hospital stays*							
0 days	Ref.	Ref.	Ref.	Ref.	Ref.		
1–6 days	3.35 (2.24–5.03)	2.51 (1.66–3.80)	2.05 (1.33–3.17)	2.55 (1.69–3.85)	2.06 (1.33–3.19)		
≥ 7 days	7.08 (4.72–10.61)	4.63 (2.99–7.19)	3.46 (2.17–5.50)	4.66 (3.00–7.23)	3.47 (2.18–5.53)		
**Co-morbidity**							
**1 previous year of the index date: OPD visits × 3, admission × 1**							
RA	18.76 (11.16–31.55)		3.37 (1.77–6.42)		3.34 (1.75–6.38)		
SS	42.89 (28.24–65.16)		7.49 (4.33–12.96)		7.45 (4.28–12.94)		
SSc	120.88 (38.75–377.04)		3.98 (1.08–14.59)		4.12 (1.10–15.42)		
Vasculitis	98.63 (36.76–264.64)		6.66 (2.11–21.07)		6.33 (1.98–20.28)		
Hypertension	1.35 (0.96–1.89)		1.42 (0.94–2.14)		1.41 (0.94–2.13)		
Diabetes mellitus	0.71 (0.38–1.33)		0.53 (0.27–1.05)		0.54 (0.27–1.05)		
Hyperlipidemia	1.08 (0.62–1.88)		0.83 (0.46–1.51)		0.82 (0.45–1.50)		
Coronary artery disease	1.28 (0.63–2.58)		0.83 (0.39–1.78)		0.83 (0.39–1.78)		
Osteoporosis	5.20 (2.99–9.07)		2.97 (1.62–5.42)		2.95 (1.62–5.39)		
Cerebral vascular accident	1.62 (0.72–3.63)		1.04 (0.44–2.47)		1.03 (0.44–2.44)		
COPD/asthma	2.00 (1.12–3.56)		1.25 (0.68–2.33)		1.24 (0.67–2.32)		
Chronic kidney disease	1.86 (0.46–7.48)		1.47 (0.36–6.05)		1.45 (0.35–5.98)		
Chronic liver diseases	3.35 (2.08–5.40)		2.34 (1.41–3.91)		2.32 (1.39–3.87)		
Pancreatitis	4.53 (0.64–32.28)		1.13 (0.15–8.80)		1.14 (0.15–8.91)		
Affective psychosis	1.42 (0.35–5.69)		0.52 (0.11–2.46)		0.53 (0.11–2.47)		
Ankylosing spondylitis	5.11 (0.72–36.31)		0.76 (0.08–7.38)		0.79 (0.08–7.57)		
IBD	0.00 (0.00–1.05E148)		0.00 (0.00–2.28E174)		0.00 (0.00–1.49E174)		
HIV infection	0.00 (0.00–2.85E155)		0.00 (0.00–9.99E999)		0.00 (0.00–9.99E999)		
AIHA	0.02 (0.00–2.33E144)		0.00 (0.00–9.99E999)		0.00 (0.00–9.99E999)		
ITP	24.38 (3.43–173.51)		8.72 (1.19–63.99)		8.79 (1.19–64.67)		

After 1:2 PSM, the HR of the conditional Cox model with HT exposure alone (model A) was 3.54 (95% CI: 2.40–5.22). In model B, the group of HT and hypothyroidism only presented the highest aHR of 4.27 (95% CI: 2.67–6.83), followed by HT, no hyperthyroidism, and no hypothyroidism: 3.47 (95% CI: 2.12–5.69) and HT, hyperthyroidism, and hypothyroidism: 3.77 (95% CI: 1.76–8.05). The least risk was HT and hyperthyroidism only, aHR: 1.72 (95% CI: 0.68–4.35), with no significant difference compared with the result before PSM. The group of the 3 months barrier for following up time shares the same results, which are presented in Table 6, but the group of 6 months barrier presents the highest aHR of 4.33 (95% CI: 2.01–9.32), presented in Table 7.

We also conducted the sensitivity analysis in the estimation of the SLE risk for HT exposure in age- and sex-matched population. In the 4 scenarios, 2 different SLE treatment plan and the exclusion of other autoimmune diseases were listed in Tables 8, 9, and 10. Under the constructive of model 3, the aHR was 4.35 (95% CI: 3.28–5.76) in scenario 1, 4.39 (95% CI: 3.31–5.82) in scenario 2, 5.11 (95% CI: 3.75–6.98) in scenario 3, and 4.70 (95% CI: 3.46–6.38) in scenario 4. The sensitivity analysis was also performed on the groups of the 3 and 6 months barrier, and all 4 scenarios in the 3 groups have high enough aHRs to support the major result.

**Table 8:** Sensitivity analysis in the estimation of the SLE risk for Hashimoto’s thyroiditis exposure in the age-matched and sex-matched population

		Model 3, aHR^a^ (95%CI)
Scenario 1	Definition of SLE event: major illness registry (main finding)	4.35 (3.28–5.76)
Scenario 2	Definition of SLE event: scenario 1 + treated with systemic corticosteroids or DMARDs (including HCQ or azathioprine)	4.39 (3.31–5.82)
Scenario 3	Definition of SLE event: scenario 1 + treated with DMARDs (including HCQ or azathioprine)^b^	5.11 (3.75–6.98)
Scenario 4	Exclusion of patients with RA, SS, SSc, vasculitis, AS, and IBD at baseline (excluding autoimmune thyroiditis accompanied with other autoimmune diseases)	4.70 (3.46–6.38)

*aHR* Adjusted HR, *HCQ* Hydroxychloroquine, *RA* Rheumatoid arthritis, *SS* Sjögren’s syndrome, *SSc* Systemic sclerosis, *AS* Ankylosing spondylitis, *IBD* Inflammatory bowel disease, *SLE* Systemic lupus erythematosus

^a^aHR: the covariates including urbanization, low income, and comorbidities listed in Table 1

^b^The treatment of SLE was identified within 6 months after the first diagnosis of SLE

**Table 9:** Sensitivity analysis in the estimation of the SLE risk for Hashimoto’s thyroiditis exposure in the age-matched and sex-matched population. Follow-up duration of samples ≧ 3 months

		Model 3, aHR^a^ (95%CI)
Scenario 1	Definition of SLE event: major illness registry (main finding)	3.84 (2.84–5.19)
Scenario 2	Definition of SLE event: scenario 1 + treated with systemic corticosteroids or DMARDs (including HCQ or azathioprine)	3.91 (2.89–5.30)
Scenario 3	Definition of SLE event: scenario 1 + treated with DMARDs (including HCQ or azathioprine)^b^	4.72 (3.41–6.55)
Scenario 4	Exclusion of patients with RA, SS, SSc, vasculitis, AS, and IBD at baseline (excluding autoimmune thyroiditis accompanied with other autoimmune diseases)	4.21 (3.06–5.79)

*aHR* adjusted HR, *HCQ* Hydroxychloroquine, *RA* Rheumatoid arthritis, *SS* Sjögren’s syndrome, *SSc* Systemic sclerosis, *AS* Ankylosing spondylitis, *IBD* Inflammatory bowel disease, *SLE* Systemic lupus erythematosus

^a^aHR: the covariates including urbanization, low income, and comorbidities listed in Table 1

^b^The treatment of SLE was identified within 6 months after the first diagnosis of SLE

**Table 10:** Sensitivity analysis in the estimation of the SLE risk for Hashimoto’s thyroiditis exposure in the age-matched and sex-matched population. Follow-up duration of samples ≧ 6 months

		Model 3, aHR^a^ (95%CI)
Scenario 1	Definition of SLE event: major illness registry (main finding)	4.13 (3.02–5.65)
Scenario 2	Definition of SLE event: scenario 1 + treated with systemic corticosteroids or DMARDs (including HCQ or azathioprine)	4.23 (3.09–5.79)
Scenario 3	Definition of SLE event: scenario 1 + treated with DMARDs (including HCQ or azathioprine)^b^	5.18 (3.70–7.26)
Scenario 4	Exclusion of patients with RA, SS, SSc, vasculitis, AS, and IBD at baseline (excluding autoimmune thyroiditis accompanied with other autoimmune diseases)	4.70 (3.39–6.51)

*aHR* Adjusted HR, *HCQ* Hydroxychloroquine, *RA* Rheumatoid arthritis, *SS* Sjögren’s syndrome, *SSc* Systemic sclerosis, *AS* Ankylosing spondylitis, *IBD* Inflammatory bowel disease, *SLE* Systemic lupus erythematosus

^a^aHR: the covariates including urbanization, low income, and comorbidities listed in Table 1

^b^The treatment of SLE was identified within 6 months after the first diagnosis of SLE

## Discussion

Although previous studies about thyroid and SLE showed that SLE patients are prone to develop hypothyroidism [2, 17], this study indeed told us that HT might also be associated with SLE (Table 11). In this population-based study in Taiwan, we found patients with a history of HT (aHR: 6.79, 95% CI: 5.32–8.66) or HT with hypothyroidism (aHR: 7.59, 95% CI: 5.34–10.81) were vulnerable to develop SLE compared to non-HT, no hyperthyroidism, and no hypothyroidism. Besides, hyperthyroidism was also a minor risk factor for SLE with a less ratio (aHR: 3.21, 95% CI: 1.43–7.21). More interestingly, in those HT patients who were combined with hyperthyroidism, the incidence of SLE decreased slightly but still higher than in the comparator group without HT. On the other hand, if HT patients once had hypothyroidism, then whether they had hyperthyroidism or not, the incidence of SLE is hardly affected compared to those with hypothyroidism only.

**Table 11:** Number at risk after PSM 1:2

Year after the beginning of this study	0	1	2	3	4	5	6	7	8	9
**No restriction on follow-up duration**										
Non-Hashimoto’s thyroiditis	31,024	30,120	25,876	21,426	17,360	13,244	9592	5947	2836	0
Hashimoto’s thyroiditis	15,512	15,325	13,217	11,040	9005	6991	5050	3165	1514	0
Hashimoto’s thyroiditis, no hyperthyroidism, and no hypothyroidism	5910	5828	4992	4177	3401	2649	1895	1176	585	0
Hashimoto’s thyroiditis and hyperthyroidism only	2367	2337	2000	1635	1297	990	674	428	194	0
Hashimoto’s thyroiditis and hypothyroidism only	5549	5480	4769	4015	3320	2624	1973	1256	593	0
Hashimoto’s thyroiditis, hyperthyroidism, and hypothyroidism	1686	1680	1456	1213	987	728	508	305	142	0
**Follow-up duration of samples ≧ 3 months**										
Non-Hashimoto’s thyroiditis	30,680	30,120	25,876	21,426	17,360	13,244	9592	5947	2836	0
Hashimoto’s thyroiditis	15,490	15,325	13,217	11,040	9005	6991	5050	3165	1514	0
Hashimoto’s thyroiditis, no hyperthyroidism, and no hypothyroidism	5900	5828	4992	4177	3401	2649	1895	1176	585	0
Hashimoto’s thyroiditis and hyperthyroidism only	2364	2337	2000	1635	1297	990	674	428	194	0
Hashimoto’s thyroiditis and hypothyroidism only	5541	5480	4769	4015	3320	2624	1973	1256	593	0
Hashimoto’s thyroiditis, hyperthyroidism, and hypothyroidism	1685	1680	1456	1213	987	728	508	305	142	0
**Follow-up duration of samples ≧ 6 months**										
Non-Hashimoto’s thyroiditis	30,524	30,120	25,876	21,426	17,360	13,244	9592	5947	2836	0
Hashimoto’s thyroiditis	15,454	15,325	13,217	11,040	9005	6991	5050	3165	1514	0
Hashimoto’s thyroiditis, no hyperthyroidism, and no hypothyroidism	5888	5828	4992	4177	3401	2649	1895	1176	585	0
Hashimoto’s thyroiditis and hyperthyroidism only	2357	2337	2000	1635	1297	990	674	428	194	0
Hashimoto’s thyroiditis and hypothyroidism only	5525	5480	4769	4015	3320	2624	1973	1256	593	0
Hashimoto’s thyroiditis, hyperthyroidism, and hypothyroidism	1684	1680	1456	1213	987	728	508	305	142	0

The reason that patients with HT are prone to develop SLE needs to be clarified. In our opinion, first, impairment of regulatory T cells (Treg) might be a key. Impaired Treg might cause the loss of self-toleration and increases the risk of autoimmune disease, including SLE [20], and according to previous studies, the loss of Treg function was found in both HT and SLE [21–23]. Second, interleukin-17 (IL-17) and Th17 which are known to participate in inflammation [24] also play important roles in autoimmune diseases, including HT and SLE [25, 26]. A study pointed out that the more IL-17 is produced by Th17, the more thyroid function is lost in HT patients [27]. As SLE shares a similar pathogenesis [26], elevated Th17 and IL-17 in HT might stimulate the progression of SLE. Third, the common presence of antinuclear antibodies (ANA) in HT patients might be a crucial factor to induce other autoimmune diseases, including SLE. A study evaluating HT patients showed that 47% of HT patients were ANA positive, and 72% of them have other autoimmunity parameters besides anti-thyroid peroxidase (anti-TPO) or antithyroglobulin (anti-Tg), and/or have an autoimmune disease besides HT [28], indicating that it is possible for HT patients to come out with other autoimmune diseases. ANA is also a highly sensitive (98%) screening marker for SLE [29]. Thus, the common presence of ANA in patients with HT might be a crucial factor to induce the development of SLE. Last, studies have shown that phagocytosis was stimulated by physiological concentrations of thyroid hormone [30], and it was decreased after thyroid suppression in an animal model [31]. It means HT patients with hypothyroidism might lose the ability to clean up the autoimmune complex and develop SLE [32], which might explain why patients with hypothyroidism have a higher risk of SLE than hyperthyroidism in our study.

The finding of HT being associated with subsequent SLE is important in clinical practice. HT is not a difficult disease to treat, however, its complications are usually forgotten, for example, thyroid lymphoma [33]. And now SLE might also be another potential complication of HT according to our study. Once SLE has been developed, it will affect the whole body from sleep disturbance [34] to pulmonary complications [35] and cardiovascular disease [36], deteriorating the health condition. Moreover, since genetic factors cannot be modified easily, it seems important to investigate the other risk factors of SLE. Early identification and intervention of SLE will minimize the deterioration and damage to the body [37].

Previous studies have told us some environmental factors causing SLE. Besides ultraviolet radiation [38], bacteria and virus infections have also been proven that they are linked to SLE by affecting the immune system [39, 40], including nontyphoidal– Salmonella [41] and Varicella zoster virus [42]. Hormones have also been investigated for their correlation with SLE. For example, estrogen can rescue autoreactive B cells from apoptosis [43], explaining why females are more prone to autoimmune disease. Progesterone can regulate CD4+ T cells [44] to prevent pregnant women from producing anti-fetal antibodies, so low levels of progesterone are considered as a predisposing factor for SLE [45]. According to many studies, thyroid hormone also can affect the immune system [10–16]. Acquiring any kind of autoimmune disease is another risk to developing another one with unclear reasons, and when there is more than three autoimmune coexistence, it is called as “multiple autoimmune syndrome (MAS)” [8, 9], and HT is classified as one of MAS [46], which might be a risk factor of developing SLE.

Despite that many studies indicated the function of thyroid hormone in the immune system [10–16] and the hypothesis about MAS increasing the risk of SLE, there are only two case reports about two young girls and two women, respectively, implying HT might be a risk factor of SLE [18, 19]. This time, we conducted a large-scale retrospective cohort study, which is more persuasive than a case report, attempting to prove this thought.

In our study, we not only surveyed the relation between HT and SLE, but also consider other factors. We have described above the thought of MAS being a factor in developing SLE, and now, our data supported it again with an extremely higher hazard ratio of SLE with other autoimmune diseases, including RA (aHR: 14.57, CI: 9.51–22.33), SS (aHR: 20.96, CI: 13.92–31.56), SSc (aHR: 61.44, CI: 19.78–190.79), vasculitis (aHR: 81.34, CI: 36.38–181.88), AIHA (aHR: 417.97, CI: 104.30–1674.87), and ITP (aHR: 67.09, CI: 27.81–161.85). On the other hand, we also found the possibility of HT alone increasing the risk of SLE by excluding other common autoimmune diseases (aHR: 4.70, CI: 3.46–6.38), which means SLE following HT might not be attributed to other autoimmune diseases.

In addition, we considered whether the status of thyroid function would affect the risk of SLE. The results showed a higher risk of SLE among patients with hypothyroidism. Despite the absolute rate being low, the increased hazard ratio of SLE gave clinical physicians a hint for caring for these HT patients. This outcome might be explained by the ability of phagocytosis of the autoimmune complex which we have mentioned above [30–32]. However, the detailed mechanism between HT and SLE still needs to be clarified.

Our study was validated enough to be a presentative of the general population by using the NHIRD of Taiwan, which has multiple advantages including a great sample size covering over 99% of nationals of Taiwan, and long-term comprehensive follow-up to assess the risk of new-onset SLE in patients with HT [47]. In addition, we performed a sensitivity analysis to confirm the conclusion of this study by using four different scenarios. In other subgroups, urbanization, low income, length of hospital stays, and other factors were examined. Gender, age, and other factors were adjusted appropriately in PSM to minimize the selection bias.

Despite the advantages mentioned above, there are still some limitations in our study. First, the use of a “major illness registry” might lead to an underestimated incidence of SLE because few patients might not get this identification. Second, smoking status, a confounder factor of SLE [48], was unavailable in the NHIRD. Hence, we used chronic obstructive pulmonary disease as a surrogate variable for cigarette smoking because of its close correlation with cigarettes [49]. Third, our study cannot explain why HT patients with hyperthyroidism had a decreased incidence of SLE despite the fact that excessive thyroid hormone can also impair Treg cells [50]. It cannot prove the pathogenesis of SLE following HT directly, either. Thus, the mechanism still needs to be investigated through more experiments. Fourth, although we have adjusted many confounders, we still missed genetic factors which are associated with both HT and SLE due to the difficulty of collecting genetic data. Last, because people recorded in NHIRD are usually Taiwanese, this study might not be applicable to non-Asian ethnic groups.

In conclusion, this population-based study suggested an increased risk of SLE in the HT group after adjustment for baseline characteristics, comorbidities, and medical confounders compared with the reference group. It could provide hints for further research to clarify the pathogenesis between HT, hypothyroidism, hyperthyroidism, and SLE.

## Data Availability

Summarized individual data are available on request to the corresponding author. The data set used in this study is managed by the Taiwan Ministry of Health and Welfare and, thus, cannot be made available publicly. Researchers interested in accessing this data set can submit a formal application to the Ministry of Health and Welfare to request access (the postal address No. 488, Section 6, Zhongxiao E Rd, Nan-gang District, Taipei City 115, Taiwan; website: https://dep.mohw.gov.tw/ DOS/cp-2516-3591-113.html).

## Abbreviations

SLE: Systemic lupus erythematosus
HT: Hashimoto’s thyroiditis
DMARDs: Disease-modifying anti-rheumatic drugs
HCQ: Hydroxychloroquine
RA: Rheumatic arthritis
SS: Sjögren’s syndrome
SSc: Systematic sclerosis
AS: Ankylosing spondylitis
IBD: Inflammatory bowel disease
COPD: Chronic obstructive pulmonary disease
CKD: Chronic kidney disease
AIHA: Autoimmune hemolytic anemia
ITP: Idiopathic thrombocytopenic purpura
ANA: Antinuclear antibody
MAS: Multiple autoimmune syndrome
